# Intraoperative Blood Loss and Postoperative Pain in the Sagittal Split Ramus Osteotomy and Intraoral Vertical Ramus Osteotomy: A Literature Review

**DOI:** 10.1155/2021/4439867

**Published:** 2021-07-03

**Authors:** Kun-Tsung Lee, Shiu-Shiung Lin, Kun-Jung Hsu, Chi-Yu Tsai, Yi-Hao Lee, Yu-Jen Chang, Te-Ju Wu

**Affiliations:** ^1^Division of Clinical Dentistry, Department of Dentistry, Kaohsiung Medical University Hospital, Kaohsiung, Taiwan; ^2^Department of Oral Hygiene, College of Dental Science, Kaohsiung Medical University, Kaohsiung, Taiwan; ^3^Department of Orthodontics, Kaohsiung Chang Gung Memorial Hospital, College of Medicine, Chang Gung University, Kaohsiung, Taiwan; ^4^Department of Dentistry, Kaohsiung Chang Gung Memorial Hospital, College of Medicine, Chang Gung University, Kaohsiung, Taiwan; ^5^Dental Department, Kaohsiung Municipal Ta-Tung Hospital, Kaohsiung, Taiwan; ^6^School of Dentistry, College of Dental Medicine, Kaohsiung Medical University, Kaohsiung, Taiwan

## Abstract

**Purpose:**

The purpose of the present study was to review the literature regarding the blood loss and postoperative pain in the isolated sagittal split ramus osteotomy (SSRO) and intraoral vertical ramus osteotomy (IVRO).

**Materials and Methods:**

Investigating the intraoperative blood loss and postoperative pain, articles were selected from 1970 to 2021 in the English published databases (PubMed, Web of Science, and Cochrane Library). Article retrieval and selection were performed by two authors, and they independently evaluated them based on the eligibility criteria. The articles meeting the search criteria had especially at least 30 patients.

**Results:**

In the review of intraoperative blood loss, a total of 139 articles were retrieved and restricted to 6 articles (SSRO: 4; IVRO: 2). In the review of postoperative pain, a total of 174 articles were retrieved and restricted to 4 articles (SSRO: 3; IVRO: 1). The mean blood loss of SSRO and IVRO was ranged from 55 to 167 mL and 82 to 104 mL, respectively. The mean visual analog scale (VAS) scores of the first postoperative day were 2 to 5.3 in SSRO and 2.93 to 3.13 in IVRO. The mean VAS scores of the second postoperative day were 1 to 3 in SSRO and 1.1 to 1.8 in IVRO.

**Conclusion:**

Compared to traditional SSRO, IVRO had a significantly lower amount of blood loss. However, the blood transfusion is not necessary in a single-jaw operation (SSRO or IVRO). Postoperative pain was similar between SSRO and IVRO.

## 1. Introduction

Orthognathic surgery has a varying level of complexity and high technical requirements. Surgeons should pay attention to other main issues, such as preoperative assessment of the patient's medical condition, duration of operation, intraoperative blood loss, degree of postoperative pain, potential postoperative sequelae, and complications. Surgeons also take into consideration the anxiety of patients. Specifically, patients worry about the potential need for blood transfusion due to intraoperative blood loss and may question the safety of various risk factors related to blood transfusion. Therefore, estimations of operation time and blood loss must be precise which is beneficial to the promotion of communication between the surgeons, anesthesiologists, patients, and their families to have sufficient understanding of the overall operation process.

Postoperative pain management is a major concern for surgical patients. Poor postoperative pain control negatively affects patient's emotions, which in turn affect postoperative quality of life and appropriate expectations of the prognosis. Sagittal split ramus osteotomy (SSRO) and intraoral vertical ramus osteotomy (IVRO) are the two most common surgical techniques for orthognathic surgery, and they vary in surgery-related variables such as operation time, blood loss, and postoperative pain. Studies [1–10] have mostly discussed the SSRO, with IVRO [11–14] being rarely addressed. The present review article conducted a literature review to compare SSRO and IVRO in terms of operation time, blood loss, and postoperative pain.

## 2. Materials and Methods

The databases (PubMed, Web of Science, and Cochrane Library) were searched for articles published in English since 1970 using the terms “sagittal split ramus osteotomy,” “intraoral vertical ramus osteotomy,” “blood loss,” and “pain.” The visual analog scale (VAS; 0, indicating no pain; 10, indicating excruciating pain) of postoperative pain was recorded. In addition, the references of the selected articles were manually searched for other relevant articles. Article retrieval and selection were performed by two authors, who then read the titles and abstracts of the studies and independently evaluated them based on the eligibility criteria. Articles meeting the criteria were selected for full-text reading. In case of a discrepancy between the authors regarding the inclusion of a study, full-text reading was chosen.

A study was included when it met the following criteria: (1) being a randomized controlled trial, case series, and observational study; (2) having at least 30 patients; and (3) involving only mandibular SSRO or IVRO. The following studies were excluded: case reports, reviews, studies involving patients with craniofacial syndromes, and studies including patients with a history of facial trauma. Demographic, methodological, intraoperative, and postoperative data were independently evaluated by two authors. Any discrepancies were resolved by discussion with other authors.

## 3. Results

A total of 96 articles were retrieved using the search terms “sagittal split ramus osteotomy” and “blood loss” in the PubMed (*n* = 66), Web of Science (*n* = 23), and Cochrane Library (*n* = 7) databases. IVRO had a total 43 articles using the search terms “intraoral vertical ramus osteotomy” and “blood loss” in the PubMed (*n* = 13), Web of Science (*n* = 23), and Cochrane Library (*n* = 7) databases. Of these, 139 articles were retained by further narrowing to 6 articles [15–20] (SSRO: 4; IVRO: 2) whose domain is in a single-mandibular operation (Table 1).

Investigating the postoperative pain, a total of 151 articles were retrieved using the search terms “sagittal split ramus osteotomy” and “pain” in the PubMed (*n* = 73), Web of Science (*n* = 55), and Cochrane Library (*n* = 23) databases. IVRO had a total of 23 articles using the search terms “intraoral vertical ramus osteotomy” and “pain” in the PubMed (*n* = 13), Web of Science (*n* = 8), and Cochrane Library (*n* = 2) databases. Of these, 174 articles were retained by further narrowing to 4 articles [21–24] (SSRO: 3; IVRO: 1) whose domain is in a single-mandibular operation (Table 2).

These studies of blood loss included a total of 350 patients (SSRO: 270; IVRO: 80). The mean operation time of SSRO and IVRO was ranged from 105 to 174 minutes and 61 to 349 minutes, respectively. The mean blood loss of SSRO and IVRO was ranged from 55 to 167 mL and 82 to 104 mL, respectively. These studies of postoperative pain included a total of 239 patients (SSRO: 197; IVRO: 42). The mean VAS scores of the first postoperative day were 2 to 5.3 in SSRO and 2.93 to 3.13 in IVRO. The mean VAS scores of the second postoperative day were 1 to 3 in SSRO and 1.1 to 1.8 in IVRO.

## 4. Discussion

Orthognathic surgery is performed to correct facial deformity, enhance masticatory function, and improve the facial appearance. Orthognathic surgical techniques must be precise to achieve the desired outcome. However, the maxillofacial region consists of complex and dense networks of blood vessels, and the view of the operation field may be limited in certain intraoral operations. Therefore, the management of surgical bleeding can sometimes be challenging. The methods for calculating blood loss had been reported as follows: (1) direct measurement: perioperative weighing of sponges and collection of suctioned fluids; (2) calculated blood loss (Nadler's formula) [25]: taking into account height, weight, and sex; (3) postoperative loss of haemoglobin and hematocrit level; (4) colorimetric blood loss estimation [26]: calculating blood loss by taking photographs of the used surgical gauze and canisters; and (5) continuous noninvasive intraoperative haemoglobin monitoring [27].

Both the methods of anesthesia [28–30] and the surgical techniques [31–33] could affect the operation time and then control the amount of blood loss. Remifentanil is an ultrashort-acting opioid that can suppress the autonomic nervous response and produce an analgesic effect. Moreover, remifentanil possesses the parasympathetic activation contributing to hemodynamic depression (bradycardia and hypotension). Twersky et al. [34] compared the hemodynamic changes using either remifentanil or fentanyl in 2,438 surgical patients. They reported that remifentanil-treated patients exhibited lower systolic and diastolic blood pressures (by 10-15 mmHg) and lower heart rates (by 10-15 bpm) intraoperatively compared to the fentanyl-treated patients. Handa et al. [18] reported that there was no significant difference between propofol-remifentanil and propofol-fentanyl for anesthesia in the mean operation time (115.8 and 112 minutes) of traditional SSRO. However, propofol-remifentanil (118.4 mL) is also significantly effective in reducing intraoperative blood loss compared to propofol-fentanyl (171.7 mL) during SSRO.

In this literature review, it was indicated that the surgical instruments used in SSRO are mainly traditional chisels and few piezoelectric devices. Shirota et al. [16] reported that there was no significant difference between traditional SSRO and piezoelectric SSRO in the operation time. However, Koba et al. [35] indicated that osteotomy time and total operation time of piezoelectric SSRO were significantly shorter than those of the traditional SSRO. Shirota et al. [16] revealed that piezoelectric SSRO did not reduce intraoperative blood loss significantly. Nonetheless, Koba et al. [35] reported a mean blood loss of only 41.6 mL in piezoelectric surgery, which differs from the findings of Shirota et al. [16] and is significantly lower than the blood loss in traditional SSRO.

Kuroyanagi et al. [15] reported a mean blood loss of only 73.3 mL in traditional SSRO, significantly lower than those measured by Shirota et al. [16] (189 mL), Handa et al. [18] (propofol-remifentanil: 118.4 mL; propofol-fentanyl: 171.7 mL, and Salma et al. (176.67 mL). This result is ascribable to the discovery by Kuroyanagi et al. [15] that a medial ramus type significantly affects operation time and blood loss. In the study of Kuroyanagi et al. [15], 59% of patients had a moderately straight medial ramus whereas the rest (41%) had a concave medial ramus. The operation time for patients with a moderately straight medial ramus was significantly shorter, and a mean blood loss of 53 mL was discovered in patients with a moderately straight medial ramus. By contrast, the patients with a concave medial ramus had a mean blood loss of 102.5 mL. Statistically, patients with a moderately straight medial ramus led to significantly less blood loss than those with a concave medial ramus. In terms of the potential correlation between blood loss and operation time, Kuroyanagi et al. [15], Shirota et al. [16], Handa et al. [18], Salma et al. [19], and Ueki et al. [12] all found a significantly positive correlation between them, whereas Böttger et al. [36] deemed the correlation between them to be weak.

In the IVRO technique, Pedersen et al. [20] reported that the mean operation time and intraoperative blood loss were 61 min and 82 mL, respectively. Chen et al. [17] found that the mean operation time and blood loss had no significant difference between female (229 minutes and 86 mL) and male (249 minutes and 104 mL). Regarding the amount of blood loss in IVRO, no significant difference was observed in Pedersen et al. [20] and Chen et al. [ 17]; however, blood loss in IVRO was significantly smaller than that in traditional SSRO. Pedersen et al. [20] and Chen et al. [ 17] founded that there were no significant correlations between operation time and blood loss. Investigating the difference of gender, Rummasak et al. [37] reported that women tend to lose more blood in orthognathic surgery than do men, whereas Salma et al. [19] found the opposite. Chen et al. [17] reported that men tend to lose more blood in IVRO than do women—concurring with the finding of Salma et al. [19] Moreover, Chen et al. [17] revealed a significantly positive correlation between blood loss and operation time that was observed in men but not in women. Mayrovitz and Regan [38] presented that facial skin perfusion in male was significantly more than that in female principally due to a larger number of perfused microvessels. Kokovic et al. [39] assessed the blood perfusion of the posterior mandible using laser Doppler flowmetry. They found that male had more blood perfusion than female. Schwaiger et al. [40] investigated the blood loss in orthognathic surgery, and male was found to be associated with significantly increased bleeding volumes in the 2-jaw surgery. Moreover, male revealed more hidden blood loss than female in SSRO. By inference, intraoperative blood loss is greater in men than in women because men have more blood vessels and higher blood perfusion. Therefore, control of bleeding takes longer in men, and the operation time is longer in male patients than in female patients.

It is an important issue regarding the necessity of intraoperative blood transfusion. Moenning et al. [3] investigated 171 patients who received SSRO and discovered that their blood loss ranged from 50 to 750 mL, amounting to a mean blood loss of 176.6 mL; none of the patients required blood transfusion. Samman et al. [4] also discovered that orthognathic surgery involving one jaw does not require blood transfusion. Numerous methods are available for preventing intraoperative blood loss and minimizing the need for blood transfusion. For example, hypotensive anesthesia [2, 5, 7] is a well-established and effective technique that has been confirmed by research to reduce 40% of blood loss during orthognathic surgery. Hypotensive anesthesia can reduce the amount of bleeding, improve visibility in the surgical field, and increase the efficiency of surgical operations and hemostasis, all of which contribute to shorter operation time, less intraoperative blood loss, and lower likelihood of needing blood transfusion. According to existing data, a mean arterial pressure between 50 and 65 mmHg is safe in healthy young patients because it does not interfere with perfusion to the brain, heart, kidneys, and liver. However, hypotensive anesthesia is safe only if physical changes in the patient are closely monitored during the operation and communication between the doctor and anesthesiologist is adequate.

Pain is a complex reaction that involves the interaction between nerve conduction and various neuroregulatory factors of the central nervous system. The postoperative pain following orthognathic surgery is not simply caused by the surgical wound. Sources of postoperative pain include damage to the lingual nerve and inferior alveolar nerve, inflammation of the surgical area, muscle stiffness and discomfort caused by the muscle and osseous tissue adapting to the postoperative area, and contraction induced by injury to the surrounding soft tissues; all of the stimuli trigger changes in the response of the central nervous system. According to the literature review [21–24], the visual analog scale (VAS) value is approximately 3 on the first day following SSRO and IVRO and drops to 1–2 on the second day. The postoperative VAS values following SSRO and IVRO are similar. Nagatsuka et al. [21], Kim et al. [22], and Raschke et al. [24] all reported a strong correlation between operation time and postoperative pain, but Chen et al. [23] found no significant correlation between them. Moreover, Chen et al. discovered that blood loss was not significantly correlated with the amount of mandibular setback and postoperative pain and that there was no gender difference in postoperative pain.

Numerous methods and techniques are available for controlling postoperative pain. Evans et al. [1] investigated 45 patients undergoing orthognathic surgery and found that no narcotic analgesics were needed to control postoperative pain in most situations. Postoperative use of nonsteroidal anti-inflammatory drugs (NSAIDs) to relieve pain or reduce morphine needs has been widely proven to be effective. According to recent research reports [41–43], patient-controlled analgesia (PCA) can control postoperative pain caused by orthognathic surgery. PCA enables patients to self-administer their medication, thereby reducing postoperative anxiety and stress, which are the main determinants of postoperative pain. PCA is proven effective at mitigating discomfort during the postoperative recovery period and significantly shortening the period of hospitalization. Our clinical experience has also indicated that NSAIDs are sufficient for controlling postoperative pain. Specifically, when NSAIDs are employed after surgery, we discovered that the VAS value reported by patients was comparable to that measured during their orthodontic treatment. This finding facilitates communication between doctors and patients before the operation, enables the patient to understand postoperative pain, and reduces the anxiety and pressure of patients facing surgery.

## 5. Conclusion

From our review, we have concluded that the administration of anesthetic drugs, medial ramus type, and selection of surgical instruments could affect the operation time and blood loss in the orthognathic surgery. Compared to traditional SSRO, IVRO had a significantly lower amount of blood loss. However, the blood transfusion is not necessary in a single-jaw operation (SSRO or IVRO). Postoperative pain was similar between SSRO and IVRO.

## Figures and Tables

**Table 1:** Demographic and study characteristics in the operation time and blood loss of the included studies.

Author	Technique	Subgroups	Age	Sex	Operation time	Blood loss	Operation time & blood loss correlation
Year		Samples	Mean (years)	F (female)	Mean (minute)	Mean (mL)	
Country of origin			Range (years)	M (male)	Range (minute)	Range (mL)	
Kuroyanagi et al.	SSRO	*n* = 50	28	F: 32	105	55	Significant
2013			17-44	M: 18	80-200	15-300	
Japan							
Shirota et al.	SSRO	Piezoelectricity (*n* = 30) Traditional SSRO (*n* = 29)	28 ± 9	F: 35	174 ± 37	189 ± 113	Significant
2014			16-49	M: 24	107-255	18-584	
Japan							
Chen et al.	IVRO	*n* = 80	F: 23.31 ± 4.06	F: 49	F: 229.39 ± 40.82	F: 86.12 ± 54.98	F: no significant
2015			M: 22.42 ± 3.73	M: 31	M: 249.52 ± 48.86	M: 104.03 ± 56.73	M: significant
Taiwan							
Handa et al.	SSRO	Propofol-fentanyl (*n* = 65) Propofol-remifentanil (*n* = 66)	26.9 ± 8.1	F: 45	112 ± 29.5	171.7 ± 130.2	NA
2016				M: 20			
Japan			25.8 ± 8.4	F: 47	115.8 ± 16.4	118.4 ± 69.6	NA
				M: 19			
Salma et al.	SSRO	*n* = 30			145.8 ± 43.8	167.67 ± 59.79	NA
2017					120-300	100-300	
Saudi Arabia							
Pedersen et al.	IVRO	*n* = 131	23.5	F: 71	61	82	No significant
2021			18-73	M: 62	31-144		
Norway							

*n*: number of samples; NA: not available.

**Table 2:** Demographic and study characteristics in the postoperative pain of the included studies.

Author	Technique	Subgroups	Age	Sex	Operation time	Postoperative pain (VAS)		
Year		Sample	Mean (years)	F (female)	Mean (minute)			
Country of origin			Range (years)	M (male)	Range (minute)	First day	Second day	Third day
Nagatsuka et al.	SSRO	Multimodal analgesia group (*n* = 41) Control group (*n* = 41)	22.4 ± 4.4	F: 25, M: 16	137.3 ± 44.9	2.5-3	2-2.5	2-2.5
2000								
Japan			20.9 ± 3.7	F: 28, M: 13	136.0 ± 43.6	2.5-3	2.5-3	2-2.5
Kim et al.	SSRO	Hegu group (*n* = 28)	27.7 ± 9.1	F: 14, M: 14	205.1 ± 30.4	2-2.5	1-1.5	0.5-1
2009		Sham group (*n* = 28)	28.6 ± 8.1	F: 13, M: 15	197.3 ± 26.5	4.5-5	2.5-3	1.5-2
Korea		Control group (*n* = 28)	29.2 ± 9.3	F: 15, M: 13	195.8 ± 29.9	4.5-5	2.5-3	1.5-2
Chen et al.	IVRO	*n* = 42	F: 24.3 ± 3.85	F: 26	F: 237.12 ± 36.75	F: 2.96 ± 1.04	F: 1.1 ± 1.16	NA
2012			M: 23.2 ± 4.7	M: 16	M: 276.25 ± 46.13	M: 3.13 ± 1.45	M: 1.8 ± 1.29	NA
Taiwan								
Raschke et al.	SSRO	*n* = 31	35.8 ± 12.8	F: 18	107.5 ± 40.4	Maximum pain	NA	NA
2017				M: 13		5.3 ± 2.5		
Germany						Minimal pain	NA	NA
						2.06 ± 1.84		

*n*: number of samples; NA: not available; VAS: visual analog scale.

## Data Availability

The data used to support the findings of this study are available from the corresponding author upon request.
